# Bone Mineral Density, Mechanical, Microstructural Properties and Mineral Content of the Femur in Growing Rats Fed with Cactus *Opuntia ficus indica* (L.) Mill. (Cactaceae) Cladodes as Calcium Source in Diet

**DOI:** 10.3390/nu9020108

**Published:** 2017-02-04

**Authors:** Ezequiel Hernández-Becerra, Elsa Gutiérrez-Cortez, Alicia Del Real, Alejandra Rojas-Molina, Mario Rodríguez-García, Efraín Rubio, Michelle Quintero-García, Isela Rojas-Molina

**Affiliations:** 1 Programa de Doctorado en Ciencias Químico Biológicas, Facultad de Química, Universidad Autónoma de Querétaro, Cerro de las Campanas S/N, C.P. 76010 Querétaro, Mexico; hernandezbecerrae@gmail.com; 2 Laboratorio de Investigación Química y Farmacológica de Productos Naturales, Facultad de Química, Universidad Autónoma de Querétaro, Cerro de las Campanas S/N, C.P. 76010 Querétaro, Mexico; rojasa@uaq.mx; 3 Facultad de Ingeniería, Licenciatura en Ingeniería Agroindustrial, Universidad Autónoma de Querétaro, Carr. a Chichimequillas s/n, C.P. 76140 Querétaro, Mexico; elsaneqpm@yahoo.com.mx; 4 FES-Cuautitlán, Universidad Nacional Autónoma de México, Laboratorio de Procesos de Transformación y Tecnologías Emergentes en Alimentos, Km 2.5 Carretera Cuautitlán–Teoloyucan, San Sebastián Xhala, Edo de México, C.P. 54714 Cuautitlán Izcalli, Mexico; 5 Departamento de Ingeniería Molecular de Materiales, Centro de Física Aplicada y Tecnología Avanzada, Universidad Nacional Autónoma de México, Campus Juriquilla, C.P. 7600 Querétaro, Mexico; adelreal@unam.mx; 6 Departamento de Nanotecnología, Centro de Física Aplicada y Tecnología Avanzada, Universidad Nacional Autónoma de México, Campus Juriquilla, C.P. 7600 Querétaro, Mexico; mariorodga@gmail.com; 7 Centro Universitario de Vinculación y Transferencia de Tecnología, Benemérita Universidad Autónoma de Puebla, Centro Universitario, Col. San Manuel S/N, C.P. 72540 Puebla, Mexico; efrainrubio@yahoo.com; 8 Programa de Maestría en Ciencias Químico Biológicas, Facultad de Química, Universidad Autónoma de Querétaro, Cerro de las Campanas S/N, C.P. 76010 Querétaro, Mexico; adr_mich@hotmail.com

**Keywords:** *Opuntia ficus indica*, cactus, maturity stage, calcium, phosphorus, microstructure, bone mineral density

## Abstract

Mechanical, microstructural properties, mineral content and bone mineral density (BMD) of the femur were evaluated in growing rats fed with *Opuntia ficus indica* (L.) Mill. (Cactaceae) cladodes at different maturity stages as calcium source. Male weanling rats were fed with cladodes at early maturity stage (25 and 60 days of age, belonging to groups N-60 and N-200, respectively) and cladodes at late maturity stage (100 and 135 days of age, belonging to groups N-400 and N-600, respectively) for 6 weeks. Additionally, a control group fed with calcium carbonate as calcium source was included for comparative purposes. All diets were fitted to the same calcium content (5 g/kg diet). The failure load of femurs was significantly lower (*p* ≤ 0.05) in groups N-60 and N-200 in comparison to N-400, N-600 and control groups. The cortical width (Ct.Wi) and trabecular thickness (Tb.Th) of the femurs in control and N-600 groups were significantly higher (*p* ≤ 0.05) than Ct.Wi and Tb.Th of femurs in groups N-60 and N-200. Trabecular separation of the femurs in N-60 and N-200 groups showed the highest values compared with all experimental groups. The highest calcium content in the femurs were observed in control, N-600 and N-400 groups; whereas the lowest phosphorus content in the bones were detected in N-200, N-600 and N-400 groups. Finally, the BMD in all experimental groups increased with age; nevertheless, the highest values were observed in N-600 and control groups during pubertal and adolescence stages. The results derived from this research demonstrate, for the first time, that the calcium found in *Opuntia ficus indica* cladodes is actually bioavailable and capable of improving mineral density and mechanical and microstructural properties of the bones. These findings suggest that the consumption of cladodes at late maturity stage within the diet might have a beneficial impact on bone health.

## 1. Introduction

For several years, populations of developing countries including Mexico and Latin America have been undergoing changes in dietary habits [1]. The nutrition transition comprising changes in food patterns and dietary intake is linked to increased energy-dense sources, such as sugars, edible oils, and processed foods, whereas consumption of complex carbohydrates, fiber, and micronutrient-rich foods including fruits and vegetables is often low. These facts, coupled with reduced physical activity, increase obesity, bone density problems and the risk of non-communicable chronic diseases among the population [2,3].

Micronutrients such as Ca, P, Mg, Sr, vitamin D and vitamin K play an important role in bone health [4,5] and are required for bone mineralization and growth. An adequate intake of calcium during childhood and adolescence is necessary to achieve the peak bone mass (PBM), which is related to the adolescent growth spurt. A low PBM is a contributing factor for determining risk of fractures in children and osteoporosis in adulthood [6]. In North America and Europe, the main dietary sources of calcium are milk and dairy products. In contrast, in Mexico and Central America the major calcium supplies within the diet are nixtamalized corn products (foodstuffs derived from the thermo-alkaline treatment of corn grains) and vegetables as a result of their low cost compared with animal products [7]. In Mexico, micronutrient deficiencies (vitamin A, folate, zinc, iron and calcium) are important public health problems, affecting the most vulnerable age groups including children between 5 and 11 years of age [8].

In plant foods, calcium bioavailability decreases due to the presence of oxalic, phytic and uronic acids. These compounds bind to calcium, forming insoluble complexes which cannot be absorbed in the intestine [9]. *Opuntia ficus indica* cladodes (modified flattened stems in cacti) are part of the usual diet of the Mexican population and represent a potential source of calcium in the human diet [10]. It has been demonstrated that calcium content in cladodes increases with maturity, while the concentration of oxalates shows a cyclic tendency [11,12]; consequently, we hypothesized that calcium bioavailability of *O. ficus indica* cladodes depends on the growth stage of the plant. This topic has not been previously investigated. Considering the above mentioned, the objective of this study was to evaluate the effect of *O. ficus indica* at different maturity stages as a calcium source within the diet on mechanical, microstructural properties, bone mineral density and mineral content of the femur in growing rats.

## 2. Methods and Materials

### 2.1. Experimental Diets

*Opuntia ficus indica* cladodes were cultivated in an experimental field in Silao, Guanajuato (Rancho Los Lorenz), México, with organic fertilizer harvested during the spring of 2015. The *O. ficus indica* cladodes at 25, 60, 100 and 135 days of maturation stage (weighing 60, 200, 400 and 600 g, respectively) were dehydrated as previously reported [10]. The experimental diets (N-60, N-200, N-400, N-600 and control) were prepared with American Institute of Nutrition Rodent Diets for growing rats (AIN-93G) [13] with modifications (Table 1) including the addition of vitamin mix (AIN-93-VX, Harlan Inc., Indianapolis, IN, USA, TD 94047) and mineral mix without calcium (AIN-93-MX, Harlan Inc., TD 04374). The calcium content in all diets was 5 g/kg diet and the calcium source in the control diet was calcium carbonate (Merck 2066, Darmstadt, Germany). Different amounts of dehydrated *O. ficus indica* at different maturity stages were added to the experimental diets as the only calcium source to achieve the aforementioned calcium content. Energy values were calculated with standard factors as follows: 4 kcal for available carbohydrates and proteins and 9 kcal for lipids.

### 2.2. Chemical Composition of Experimental Diets

Control and experimental diets were studied. Moisture, ashes, total lipids and crude protein (*N* × 6.25) were measured as shown in Table 2. In addition, the total carbohydrate content was analyzed by difference (% carbohydrates = 100 − % moisture − % proteins − % lipids − % minerals) [14]. Quantifications of calcium (Ca), phosphorus (P) magnesium (Mg) and potassium (K) of the diets were carried out by inductively coupled plasma mass spectrometry (ICP-MS) and using ICP-EOS Variant 730-ES (Kyoto, Japan) equipment. All tests were performed following the official methods [15].

### 2.3. Experimental Design

Thirty-five 4-week-old male Wistar rats were obtained from the Animal Production Unit (Neurobiology Institute of the National Autonomous University of Mexico), and were housed separately in five groups (*n* = 7 per group). After a week of adaptation, the animals were separately housed in metabolic cages and kept at a temperature- (22 °C ± 2 °C) and light-controlled (12-h day–night cycle) room. The study was approved by the Autonomous University of Queretaro, Natural Sciences Department, Bioethics Committee. The animals had ad libitum access to deionized water and diets for 6 weeks. The food intake was recorded daily and the body weights were measured every week. The food efficiency was determined by the relationship of the weight gain and the food intake at the end of the experiment [16].

### 2.4. Sampling Procedures

**Figure 1:**
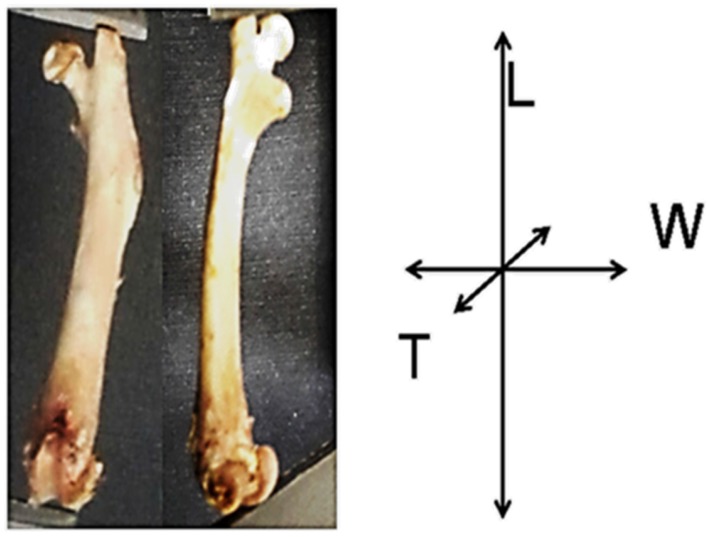
Images of the left femur of growing rats showing anterior and lateral views. Coordinates indicate length (L), width (W) and thickness (T).

At the end of the experimental period, the animals were fasted for 12 h and then killed by prolonged ethyl ether inhalation. The femurs were separated with blade surgery, eliminating the tissue adhering to the bone. The fresh and dry femurs were weighed and measured using a Vernier (Absolute Digimatic, Mitutoyo, Japan). Length measures were made, and the width and thickness were measured at the midpoint (diaphysis) of the femurs (see Figure 1). Subsequently, the femurs were labeled and stored at −20 °C until the time of their analysis.

### 2.5. Mechanical Testing

Mechanical properties of the right femurs were analyzed on a material testing machine (Zwick/Roell, Ulm, Germany, Mod. Z005, load cell 5000 N), employing the TestXpert Intelligent testing version 12.0 software. The samples were defrosted and all the measurements were carried out at room temperature. The failure load of femurs was evaluated by three-point bending (maximum breaking force of failure when the load is applied in a perpendicular plane to the longitudinal axis of the femur, denoted by the symbol *P*_max_) and compression tests (maximum force of failure when the force is applied in a vertical plane to the longitudinal axis of the femur, denoted by the symbol *F*_max_). Additionally, Young’s modulus or elastic modulus (*E*) was calculated. All the tests were evaluated in the mid-diaphyseal region of the femur [17,18].

### 2.6. Analysis of Microstructural Parameters by Scanning Electron Microscopy (SEM)

Microstructural parameters of femurs such as trabecular separation (Tb.Sp), trabecular thickness (Tb.Th) and cortical width (Ct.Wi) were analyzed in a scanning electron microscope (Jeol JSM 6060LV, Tokyo, Japan). Prior to the analysis, the left femurs were exposed at 130 °C in a Papin reactor (Cinsa, Mexico City, Mexico, 6 L) for 1 h, and thereafter, the bones were dried in an oven (Felisa, Zapopan, Jal., Mexico, FE-295A) at 60 °C to a constant weight. Subsequently, the femurs were each cut longitudinally from the intercondylar line to the diaphysis with a disc diamond saw (Diaflex-transvident 350–352, Berlin, Germany) according to previous reports [19] with some modifications. Organic compounds were removed according to a previously reported methodology [20] with modifications. The femur sections were incubated with protease (Sigma Chemical Co., St. Louis, MO, USA, P-6911; 1% *w*/*v*) at 37 °C for 24 h. Bones were subjected to a second digestion process with aminopeptidase (Sigma Chemical Co., P-6887; 0.4% *w*/*v*) at 37 °C for 18 h. Then, lipids in bones were removed with ethyl ether (J.T. Baker 9240-03, Center Valley, PA, USA) and acetone (J.T. Baker 9006-03) on a shaker (Daigger^®^ AR-100, Vernon Hills, IL, USA) for 12 h. The femurs were dried at 60 °C to a constant weight. Finally, the bones were mounted on stubs and coated with gold using an ion sputter for observation.

### 2.7. Bone Mineral Content

The left femurs were used for mineral analyses. Ca, P, Mg and K levels were determined by ICP-MS as was previously described for the mineral analyses of the diets.

### 2.8. Bone Mineral Density (BMD) Measurements

Bone mineral density of the experimental rats was registered weekly. The animal’s right femurs were scanned using a single X-ray instrument (Satelec X-mind^®^, Cologne, France) with a potential of 70 kV, a current of 8 mA, and a wavelength of 0.177 × 10^−10^ m (Toshiba X-ray tube DG-073B-DC, Tokyo, Japan) employing a Complementary metal-oxide semiconductor (CMOS) image sensor (CMOS sensor, Hamamatsu, Iwata, Japan, S10835) and a computer platform as previously described [21].

### 2.9. Statistical Analyses

The results are expressed as mean values and standard deviation (SD). The data were analyzed using one-way analysis of variance (ANOVA) followed by Tukey’s test with α = 0.05 and using the Statgraphics procedure (Graphics Software System, Manugistics Inc., Rockville, MD, USA). Furthermore, BMD, mechanical, microstructural properties and mineral content of the femur in all groups were analyzed by Pearson correlation (*r*) in order to find possible interrelationships.

## 3. Results

### 3.1. Mineral Content in O. ficus Indica at Different Maturity Stages

The average magnesium, potassium, calcium and phosphorus content in *O. ficus indica* at different maturity stages are presented in Table 3. It is quite clear that the calcium content in the cladodes increases as maturity progresses. The calcium content in the cladodes with a weight of 200, 400 and 600 g increases 97%, and 103% respectively, in comparison to the cladodes with a weight of 60 g, with significant differences (*p* ≤ 0.05). In contrast, the potassium content significantly decreased (*p* ≤ 0.05) in older cladodes. On the other hand, magnesium content did not increase as much as calcium with cladode age. Phosphorus content did not show a clear relationship with the maturity stage.

### 3.2. Chemical Composition of Experimental Diets

The chemical composition of control and experimental diets is shown in Table 2. The energy content of diets was distributed as follows: 63%, 20% and 17% (approximately) corresponding to carbohydrates, proteins and lipids, respectively. The energy density of diets ranged from 3600 to 3700 kcal/kg diet. This is in accordance with the growing rodent recommendations [13]. The calcium/phosphorus ratio in all the experimental diets ranged from 0.93 to 1.23. The lowest calcium/phosphorus ratio was observed in diets N-60 and N-200 (0.93 and 0.99, respectively). The highest calcium/phosphorus ratio was detected in diet N-400 (1.23). According to previous studies, animals fed with diets that had a Ca/P ratio in a range from 1 to 1.4 did not show negative effects on mechanical properties and microarchitecture of bones of growing rats [22,23].

### 3.3. Body Weight Gain, Food Intake and Food Efficiency in Rats Fed with Control and Experimental Diets

*O. ficus indica* significantly affected (*p* ≤ 0.05) the overall diet consumption in the experimental groups compared to the control group as is shown in Table 4. The group fed with the N-600 diet consumed the least amount of food per rat (926 ± 21.1 g), while the control group consumed more food per rat (1075 ± 53.2 g). The average body weight gain in all groups at the end of the experimental period showed no significant differences (*p* ≤ 0.05). The group fed with N-60 showed the lowest average weight gain (192.7 ± 222 g) and the group fed with N-600 showed the highest value (221.2 ± 30 g). The maximum food efficiency values were observed in groups fed with N-200 and N-600.

### 3.4. Assessment of Physical and Mechanical Properties of Femur in Rats Fed with O. ficus indica as Calcium Source

The physical and mechanical properties of femur of rats fed with experimental diets are shown in Table 5. No significant differences (*p* ≤ 0.05) were found in the mean values of length and width of the femur bones of rats fed with experimental diets. On the other hand, the femurs of rats in the experimental groups that consumed diets with cladodes at late maturity stage (N-400 and N-600) displayed the highest values of weight and thickness compared to the femurs of rats fed with cladodes at early maturity stage (N-60 y N-200). Significant differences (*p* ≤ 0.05) were found between the weight and thickness of the femurs of rats fed with the N-60 diet and those of the femurs of rats fed with the N-600 diet. Regarding femoral mechanical properties of the femur in rats of experimental groups, the lowest values derived from the compression and the three point bending tests (*F*_max_ and *P*_max_, respectively) were obtained in bone samples from the test groups fed with N-60 and N-200 diets (Table 5). Additionally, the *P*_max_ values in the test groups fed with N-60 and N-200 diets were significantly different (*p* ≤ 0.05) in comparison to the test groups fed with N-400 and N-600 diets. It is noteworthy that no significant difference (*p* ≤ 0.05) in *P*_max_ values between the test group fed with N-600 diet and control group was observed. With respect to the compression test, the data trend is the same that of the values obtained in the three-point bending test. This means that higher *F*_max_ values were detected in the femurs of test groups fed with N-400 and N-600 diets in comparison to N-60 and N-200 test groups. The *F*_max_ values in these groups decreased 23.6% and 18.5% respectively, in comparison to the control group. No significant difference (*p* ≤ 0.05) in *F*_max_ was found between the N-400 test group and the control group. In contrast, this value in the N-600 test group was significantly (*p* ≤ 0.05) higher than that observed in the control group (Table 5). Young’s modulus (*E*) of the femur in rats of test groups is shown in Table 5. The lowest *E* value was detected in the control group (825 ± 78 N/mm^2^) in comparison to the test groups fed with N-60, N-400 and N-600 diets (2554 ± 283, 2176 ± 367 and 2700 ± 194 N/mm^2^, respectively); while the control and test group fed with N-200 diet showed the lowest values (825 ± 78 and 1304 ± 230 N/mm^2^, respectively). No significant differences (*p* ≤ 0.05) were observed in these groups.

### 3.5. Assessment of Microstructural Properties of Femur in Rats Fed with O. ficus indica as Calcium Source by SEM

**Figure 2:**
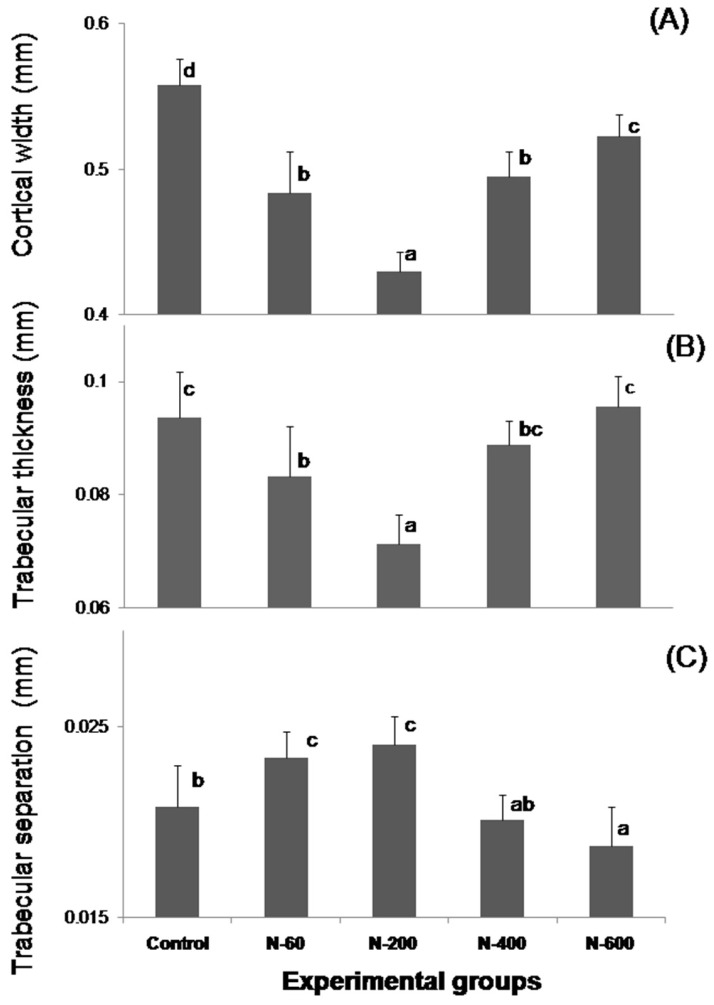
Microstructural parameters of the femur of male Wistar growing rats fed with *O. ficus indica* at different maturity stages as dietary calcium source. (**A**) Cortical width of femoral diaphysis (Ct.Wi); (**B**) trabecular thickness of femoral metaphysis (Tb.Th); (**C**) trabecular separation of femoral epiphyseal line growth (Tb.Sp). The values represent mean ± SD *n* = 7 Means in bars with different letters differ significantly (*p* ≤ 0.05).

The microstructural properties of the rat’s femurs are shown on Figure 2. Significant variations (*p* ≤ 0.05) in the cortical width (Ct.Wi) of femoral diaphysis in all groups are evident (Figure 2A). All the animals fed with *O. ficus indica* as calcium source within the diet had significantly lower Ct.Wi than the control group (0.56 ± 2 × 10^−2^ mm). The group fed with N-600 diet recorded the highest value of Ct.Wi (0.52 ± 5 × 10^−3^ mm); whereas the group fed with N-200 recorded the lowest value (0.43 ± 6 × 10^−3^mm).

Figure 2B displays a trabecular thickness (Tb.Th) of femoral metaphysis. The highest Tb.Th values were observed in the femurs of rats fed N-600 diet and the control group (0.096 ± 1 × 10^−2^ and 0.094 ± 6 × 10^−3^ mm, respectively), no significant differences were detected between these groups (*P* ≤ 0.05). In contrast, the femurs of rats fed with N-200 and N-60 showed the lowest Tb.Th values (0.071 ± 5 × 10^−3^ and 0.083 ± 8 × 10^−3^ mm, respectively) with significant differences (*p* ≤ 0.05) compared with rats fed with control and N-600 diets. Figure 2C shows the trabecular separation (Tb.Sp) of femoral epiphyseal line growth. The animals fed with *O. ficus indica* at early maturity stages (N-60 and N-200 diets) had the highest Tb.Sp values in comparison to the animals fed with *O. ficus indica* at late maturity stages (N-400 and N-600 diets) and control group. It is important to denote that Tb.Sp in rats fed with N-600 diet was significantly lower than Tb.Sp in the control group (*p* ≤ 0.05).

**Figure 3:**
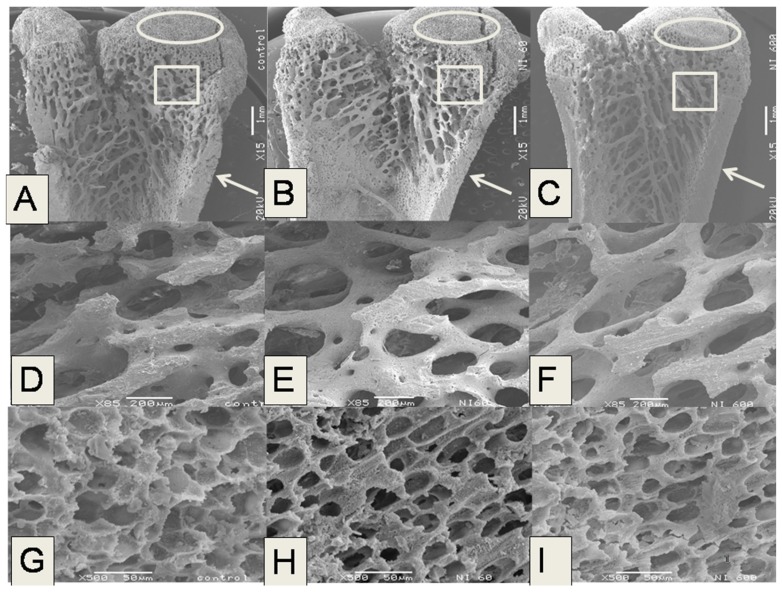
Scanning electron microscopy images of different areas of the inside left femur of male Wistar growing rats fed with *O. ficus indica* at different maturity stages as dietary calcium source. Left to right: Control, N-60 and N-600 groups. Micrographs (**A**–**C**) show the inner part of the femur sectioned longitudinally from the line between condyles toward the diaphysis (15×); Micrographs (**D**–**F**) show the femoral metaphysis (85×); Micrographs (**G**–**I**) shows the femoral epiphyseal line growth (500×).

Figure 3 shows micrographs of the distal left femoral metaphysis of rats fed with control and experimental diets. This region was used to analyze the microstructural properties of bones in cortical and trabecular (cancellous) tissues. The white arrows point the area used to measure the Ct.Wi. The squares indicate the area used to determine Tb.Th and matches with Figure 3D–F. Finally, the ovals show a region of epiphyseal growth line, where Tb.Sp was evaluated and matches with Figure 3G–I. It is important to mention that cortical bone notably contributes to the mechanical properties of the whole bone, such as resistance to fracture, whereas trabecular bone is a tissue that has important metabolic activity related with calcium homeostasis within the blood and physiological activities of this mineral. Hence, the changes in cortical and trabecular tissues are closely linked to the mechanical properties of the bones. For comparison purposes, Figure 3 shows electron microscopy images of the inner part of femurs obtained from control animals and those belonging to the N-60, and N-600 groups, which displayed the lowest and highest values for Ct.Wi, Tb.Th and Tb.Sp, respectively. These micrographs evidenced that the femoral cortical bone (at the level of the distal metaphysis) of rats fed with N-60 diet is thinner than that of femurs obtained from rats fed with control and N-600 diets (Figure 3A–C, see arrows).

Likewise, the trabecular bone area (TbAr) in the femurs of rats fed with N-60 is less widespread (Figure 3B) compared to TbAr observed in the femurs of the other experimental groups (Figure 3A,C). Figure 3D–F show femur metaphysis detail. In these figures it is noticeable that trabeculae in group fed with N-60 diet displayed high porosity, whereas the groups fed with control and N-600 diets displayed lower porosity. Finally, Figure 3G–I displays a segment of the growth zone in the femoral epiphysis of bones in rats fed with N-60 and N-600 diets compared with the control diet. It is noticeable that trabeculae of the rats fed with N-60 are less mineralized in comparison to the trabeculae of rats fed with N-600 and control diets.

### 3.6. Assessment of Bone Mineral Content

Table 6 exhibits mineral content in the femurs of rats fed with *O. ficus indica* as a calcium source and control diet. Significant differences (*p* ≤ 0.05) in the Ca content were detected between the groups. The control group displayed the highest value of Ca content, while Ca content in the groups fed with N-60, N-200, N-400 and N-600 diets decreased by 50%, 25%, 13%, and 7.5% respectively, compared to the control group. In relation to the P content, the groups fed with the control and N-60 diets showed the highest values, these groups were not different significantly (*p* ≤ 0.05). In contrast, groups fed with N-200, N-400 and N-600 diets exhibited the lowest values. As a result of these data, the calcium/phosphorus ratio of the femurs in experimental groups was affected by the maturity stage of cladodes used to prepare the diets. The maximum Ca/P ratio values were observed in the groups fed with cladodes at later maturity stages (N-400 and N-600 diets) and the control group; while the lowest Ca/P ratio values were detected in the groups fed with cladodes at early maturity stages (N-60 and N-200 diets). The relationship between the Ca/P ratio in diets and Ca/P ratio in the femurs in all groups was *r* = 0.82 (*p* ≤ 0.01). In the same way, Ca/P ratio in the femurs were related with failure load, with *P*_max_ *r* = 0.91 (*p* ≤ 0.01) and *F*_max_ *r* = 0.75 (*p* ≤ 0.01).

There were significant changes in magnesium and potassium levels in the bones of experimental groups; nonetheless, these variations were not related to the maturity stage of cladodes. The rats fed with N-60 and N-400 diets displayed the highest levels of bone magnesium, whereas the femurs of the rats fed with N-60 diet exhibited the lowest potassium content. The groups fed with N-200, N-400 and N-600 demonstrated the highest potassium content in comparison to the control group.

### 3.7. Assessment of Bone Mineral Density

**Figure 4:**
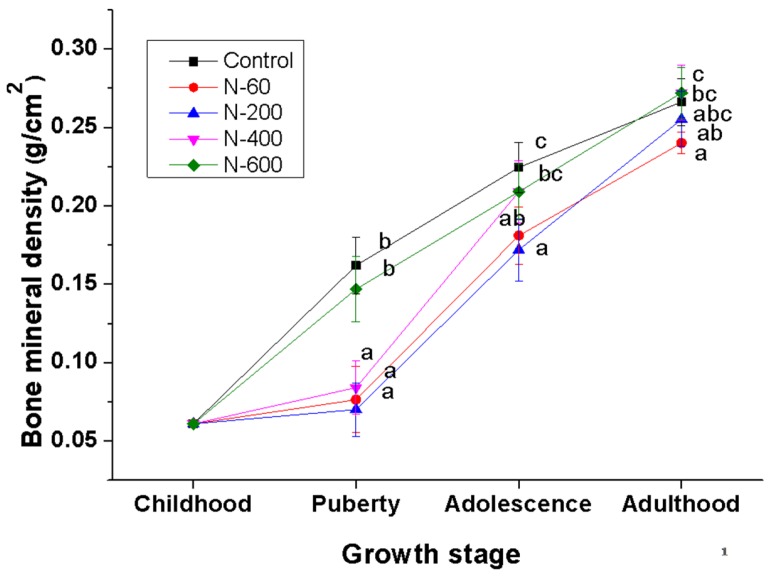
Bone mineral density (BMD) of the right femur of male Wistar growing rats fed with *O. ficus indica* at different maturity stages as dietary calcium source. Values represent mean ± SD *n* = 7; Means in lines with different letters differ significantly (*p* ≤ 0.05).

Figure 4 shows that BMD in the femur increases with the development stage of the rats in all experimental groups.

In the groups fed with control and N-600 diets a further increase was observed in BMD during puberty, while in groups fed with N-60, N-200 and N-400 diets the largest increase in BMD was detected during adolescence. It is important to denote that in the groups fed with control and N-600 diets BMD was significantly higher (*p* ≤ 0.05) in adolescence and puberty in comparison to the groups fed with N-60, N-200 and N-400. In adulthood no significant differences (*p* ≤ 0.05) in BMD were observed in the control and N-600 groups. In contrast, at this stage BMD in the N-60, N-200 groups was significantly lower compared to the control and N-600 groups.

Table 7 shows a summary of *r* values between BMD, mechanical, microstructural properties and mineral content of femurs in all groups. Prior to these analyses, a normal distribution check was carried out on all data. Strong correlations were found between Pmax, Fmax, Ca and K content, as well as Ca/P ratio and BMD (*r* ≥ 0.5). Moderated correlations were observed between microstructural properties and BMD (*r* ≤ 0.5).

## 4. Discussion

Although calcium and phosphorus are the main minerals related to bone health, minerals such as potassium and magnesium have gained importance recently since greater intakes of these micronutrients are associated with lower declines in BMD [24,25]. The results derived from this study agree with previous reports, which indicate that late maturity stage cladodes of *O. ficus indica* have high levels of calcium, phosphorus, potassium and magnesium, [11]. This suggests that their consumption in diet might help the maintenance of BMD. All experimental groups fed with *O. ficus indica* as the only calcium source consumed less food compared with the control group. This result can be attributed to the satiating effect induced by *O. ficus indica* fiber content, whose composition changes as a function of the plant age [11,26,27]. In this study, the groups fed with N-200 an N-600 diet showed higher food efficiency compared with the control group. It is well documented that dietary fiber cannot be digested in the small intestine, while in the large intestine this fiber is fermented, producing a short-chain fatty acid that can be reabsorbed into the bloodstream and increase the caloric potential of experimental diets prepared with cactus *O. ficus indica* [27,28]. Food efficiency in terms of weight gain (g)/food intake (g) ratio values found in this work are similar to those reported by Lobo et al. [16] for experimental animals of the same age. However, once adulthood is reached, the food efficiency diminishes by nearly 50%, this fact can be ascribed to the growing reduction (weight and size), whereas the food consumption at this stage remained constant. Differences in weight and thickness of femurs in the group fed with N-600 diet in comparison to bones in the group fed with the N-60 diet can be a result of variations in mineral content in *O. ficus indica* at different maturity stages (see Table 3), specifically, Mg and K, which contribute to bone formation [24,25,29]. Furthermore, identification of CaHCO_3_, CaCO_3_, CaMg(CO_3_)_2_ and MgO (bioavailable for the human body as source of Ca and Mg) in cladodes at late maturity stages [30] support these results. In addition, high calcium oxalate content in cladodes at early maturity stages reported previously [11] hinder calcium bioavailability [31]. Under long-term low levels of calcium intake, the bone supplies calcium through the process of bone resorption, which explains less thickening and reduced bone mass gain to maintain homeostasis of the organism resulting in bone loss [32].

As expected, considering greater dimensions of femurs (weight and thickness), the mechanical parameters (*F*_max_, *P*_max_ and *E*) of the femurs in groups fed with cladodes at late maturity stages (N-400 and N-600) were higher than in groups fed with cladodes at early maturity stages (N-60 and N-200). These findings might be partly ascribed to the higher levels of oxalates contained in N-60 and N-200 diets, which impair calcium absorption. Under normal conditions, the hydroxyapatite (HAP) molecules are vertically oriented to the longitudinal axis of the femur to maximize bone resistance [33]. It is widely reported that magnesium competes with calcium for the active growth sites of HAP crystals [34]. It has been demonstrated that when magnesium competes with calcium for the binding sites in HAP, crystal lattices are modified, which consequently influences the mechanical properties of bones [35]. Nevertheless, even when significant differences were observed in the magnesium content; modifications in the load to failure of bones can be attributed to the calcium content and the Ca/P ratio in the femurs as it is explained below [36].

The microstructural analysis of the femurs in the experimental groups fed with N-60 and N-200 demonstrated that these bones showed lower trabecular bone areas, Ct.Wi and Tb.Th than femurs of rats fed with control, N-400 and N-600 diets. The trabecular bone located at the ends of the femur contributes to absorb and distribute the loads applied at a point along this bone [20,37]. Therefore, lower trabecular surface areas have been linked to an increase in fracture risk and osteoporosis. In addition, some authors have found that significant bone loss is more evident in the trabecular bone compartments, since this type of bone tissue is the metabolically most active compartment of the skeleton [38]. In the case of the present study the most significant changes in microstructure were observed in trabecular and cortical bones. Figure 2 shows that N-200 was the least effective sample in providing absorbable calcium, this is probably due to a combination of several factors that include: (a) a lower level of soluble dietary fiber (SDF), which is known to facilitate calcium absorption; and (b) a variation in content of bioavailable calcium salts, which are associated to SDF, as was previously reported by our research group [10,11]. Microstructural characteristics of the femurs were positively related to the mechanical properties. These findings are in accordance with previous reports stating that osteon morphology has an important influence on facture resistance of cortical bone [39]. Recently, polyphenols and flavonoids were found in *O. ficus indica* cladodes, the content of these phytochemicals modifies at different ages [40]. Regarding this, polyphenols protect bone health through modulation of osteoblastogenesis, osteoclastogenesis and osteoimmunological action [41]. Undoubtedly, variations of chemical components (oxalates, polyphenols and flavonoids) in *O. ficus indica* at different maturity stages are responsible for the differences in calcium bioavailability and consequently, within the bone’s properties.

The mineral content in the bone, specifically Ca and P, constitute a widely accepted criterion for assessing bone health [22,36]. According to Table 6, the bones of rats fed with *O. ficus indica* at late development stage (N-400 and N-600) had higher calcium content than the bones of rats fed with *O. ficus indica* at early development stage. Taking into account that the calcium content in all diets was adjusted to the same concentration (5 g /kg diet), the differences found in the calcium content in the femurs of experimental groups fed with cladodes at different development stages can be explained by chemical composition of *O. ficus indica* used in the preparation of diets, specifically reduced anti-nutritional compounds content (oxalates) [12,31], as well as the formation of crystalline compounds (calcium carbonate, calcium-magnesium bicarbonate and magnesium oxide, etc.) in the cladodes at late development stages [10,30]. It is important to note that these crystalline compounds are associated with beneficial effects on bone health [24,42]. Moreover, it has been reported that dietary fiber composition influences short-chain fatty acid (SCFA) formation through fiber fermentation caused by colonic bacteria in the large intestine. The SCFAs lower the intestinal pH, which in turn dissolves insoluble mineral salts, particularly calcium, magnesium and iron, increasing their absorption [16,43,44,45].

P and Ca are the most studied minerals to assess the bone health [46]. Likewise, monitoring the relationship between calcium and phosphorus (Ca/P ratio) in the diet helps to detect changes that occur in normal and diseased bone due to increased calcium intake, as well as high dietary calcium/phosphorus ratios which have favorable effects on bone mass [47]. Low values of Ca/P ratio within the bones of rats fed with N-60 diet, as well as high values of this ratio in the bones of rats fed with control and N-600 diets demonstrate that the Ca/P ratio in the diet is strongly associated with Ca/P ratio in bone. These results confirm that the bone Ca/P ratio is a good index of bone quality, as has been reported previously [48]. Regarding this, other researchers have suggested that a very low dietary Ca/P ratio (0.25) triggers high concentrations of serum parathyroid hormone (PHT) persistently, which increases the bone’s resorption and hence turnover; this physiological adaptation probably holds at any life stage [46,49].

The minerals found in trace amounts in the skeleton, such as magnesium and potassium, also play an important role in the bone health [24,25]. Regarding the magnesium content in the bones it is interesting to note that even when the Mg content in diets prepared with *O. ficus indica* increased from 2.3 up to 3.5 times compared to the control diet, the magnesium content in bones of experimental animals was (*p* ≤ 0.05) lower than or equal to the control group. These results are in accordance with previous reports related to the magnesium consumption, where a high Mg intake (2–5 times Mg requirements) in growing rats had no effect on Mg content in tibia in short term studies (4 weeks) [50]. Modifications in macro-structural parameters of bones in growing rats (mid-lateral diameters and the ratios dry weight/length of humeri) have been observed in extreme conditions, such as long term studies (7 months) and excessive Mg supplementation (4-fold Mg requirements) [51]. At this point, it is important to denote that the effect of Mg in bone health has not been fully clarified, since some researchers have reported that magnesium supplementation (3 times Mg requirements) reduces calcium retention in growing male rats as a result of Mg inhibitory effect on calcium absorption in intestine, and promotes the secretion of endogenous calcium into intestine [52]; while other studies have revealed that Mg supplementation promotes bone formation, prevents bone resorption and increases the dynamic strength of the bones in ovariectomized (OVX) rats [53].

On the other hand, concerning potassium, this study showed that different K content in diets containing *O. ficus indica* (from 2.3 to 4.9 times compared to the control diet) affected content of this mineral within the bones of experimental groups; although the K consumption in diets was not correlated with the K content in femurs. In this regard, it has been demonstrated in long-term studies that high dietary potassium intake shows positive association with bone density in elderly women, suggesting that increasing the consumption of food rich in potassium may play a role in osteoporosis prevention [54]. It has been shown that supplementation with alkaline potassium salts such as potassium bicarbonate (KHCO_3_), potassium citrate (C_6_H_5_K_3_O_7_) and potassium chloride (KCl) leads to significant reduction in renal calcium excretion since potassium promotes an alkaline environment and reduces bone resorption in humans [25,55,56,57]. Regarding this, it is worth mentioning that KCl has been previously identified in *O. ficus indica* cladodes [30].

The evaluation of BMD is widely accepted to diagnose bone health and to assess the effectiveness of treatments for skeletal diseases [58,59]. The groups fed with the control and N-600 diets showed the highest values of BMD and in both groups the gain of BMD was fastest in the pubertal stage. This effect may be related, as already mentioned above, with a lower concentration of oxalates [12], and the presence of phytochemicals i.e., isoflavones and phytosterols in cladodes, which influence the bone mass gain trough the regulation of the bone cells (osteoblasts and osteoclasts) responsible of bone formation and resorption, respectively [60,61].

On the other hand, the groups fed with N-60, N-200 and N-400 diets showed an abrupt increase in BMD at the adolescence stage. This increase can be explained by adaptation process of experimental animals to dietary fiber consumption, as well as to the fiber composition, which, in the case of *O. ficus indica*, is modified depending on the maturity stage, as previously mentioned.

Finally, the results derived from this research revealed a strong correlation between the mechanical properties and BMD. These results are in accordance with previous findings stating that prior knowledge of cancellous bone density is a reasonable prediction of mechanical stiffness and strength of bone [62]. The results from this study have also demonstrated that along with mechanical properties, the microstructural parameters (Ct.Wi, Tb.Wi and Tb.Sp) are correlated with BMD, as previously reported [17,39]. A significant finding in this research is that Ca and K content in bone are correlated with BMD. In the case of calcium, this is due to calcium intake through diet or supplements increasing bone density and retarding the loss of bone stock [63]. With respect to potassium some authors suggest that potassium intake has a beneficial effect on bone mass in adult men, elderly men and women, adult men and peri- and early post-menopausal women [61]. Probably, this is due to an anion-independent effect of potassium on calcium excretion and bone metabolism [64].

## 5. Conclusions

The results in this study show evidence that *O. ficus indica* at different maturity stages have different effects on bone properties of rats in growing stage. Cladodes at early maturity stage do not provide an adequate source of dietary calcium, as was demonstrated by the detrimental effects in the femur properties in rats fed with *O ficus indica* cladodes with 60 and 200 g weight. In contrast, cladodes with 400 and 600 g weight showed the greatest potential as a calcium source within their diet, supported by the physical, mechanical, and microstructural properties, mineral content and bone mineral density in the femurs of rats fed with cladodes at the late maturity stage. The results supported by this research demonstrate, for the first time, that the calcium contained in *O. ficus indica* cladodes is actually bioavailable and capable of improving the mineral density and mechanical and microstructural properties of bones in rats. These findings suggest that the consumption of cladodes at the late maturity stage in the diet might have a beneficial impact on bone health. The effects of consumption of *O. ficus indica* on human bone health need to be further evaluated.

## Figures and Tables

**Table 1:** Ingredient composition of the experimental diets with *Opuntia ficus indica* as calcium source.

Ingredientes	Control (g/kg)	N-60 ^a^ (g/kg)	N-200 ^a^ (g/kg)	N-400 ^a^ (g/kg)	N-600 ^a^ (g/kg)
Corn starch	530	517	519	520	521
Sucrose	100	100	100	100	100
Casein ^b^	200	177	193	195.6	195
Soybean oil	70	63.9	66	68	67.5
Fiber ^c^	50	30	36.8	37	38
MixMin ^d^	49	34	42	44	43
Mix Vit ^e^	*10*	10	10	10	10
L-Cystein	3	3	3	3	3
Choline bitartarate	2.5	2.5	2.5	2.5	2.5
CaCO_3_^f^	12.5	-	-	-	-
*O. ficus indica* ^g^	-	280	168	140	132

^a^ Diets prepared with cladodes of 60 g (N-60), 200 g (N-200), 400 g (N-400) and 600 g (N-600) of weight; ^b^ Casein Sigma Chemical, Inc., St. Louis, MO, USA, C-7078; ^c^ α-Cell Fiber Solft Zolca MPbiomedical, Santa Ana, CA, USA. In diets prepared with *O. ficus indica*, the fiber content was fitted to 50 g/kg diet, taking into consideration the fiber content in cladodes; ^d^ Mineral mix without calcium (AIN-93-MX, Harlan Inc., Indianapolis, IN, USA, TD 04374) [13]; ^e^ Vitamin mix (AIN-93-VX, Harlan Inc., TD 94047) [13]; ^f^ Control diet contained CaCO_3_ (Merck 2066, Darmstadt, Germany) as calcium source; ^g^ In experimental diets, *O. ficus indica* powder provided 5 g/kg of calcium, as well as the carbohydrates, proteins and lipids, that complemented nutritional requirements in experimental diets (AIN-93G) [13].

**Table 2:** Chemical composition of control and experimental diets.

Content (%)	Control	N-60	N-200	N-400	N-600
Moisture	8.00	8.56	7.95	7.70	7.20
Ash	2.10	1.64	2.25	2.40	2.60
Carbohydrates ^a^	63.90	63.20	63.40	63.50	62.70
Protein ^a^	19.50	19.90	19.80	19.80	20.20
Lipids ^a^	16.50	16.70	16.60	16.60	17.30
Kcal/kg diet	3777	3735	3738	3741	3668
Magnesium	0.05	0.25	0.18	0.16	0.15
Potassium	0.36	1.65	1.02	0.82	0.81
Calcium	0.47	0.45	0.44	0.51	0.48
Phosphorus	0.29	0.37	0.34	0.32	0.33
Ca/P ratio	1.25	0.93	0.99	1.23	1.15

^a^ The values represent caloric percentage contribution of carbohydrates, proteins and lipids in diets.

**Table 3:** Mineral content in *Opuntia ficus índica* cladodes at different maturity stages (mg/g).

Maturity Stage (Days)	Calcium	Phosphorus	Potassium	Magnesium
25	17.2 ± 0.05 ^a^	2.9 ± 0.01 ^a^	46.3 ± 0.14 ^a^	7.2 ± 0.31 ^a^
60	29.3 ± 0.04 ^b^	2.2 ± 0.01 ^b^	39.2 ± 0.15 ^c^	7.4 ± 0.21 ^b^
100	33.5 ± 0.06 ^c^	1.7 ± 0.01 ^c^	33.1 ± 0.14 ^b^	7.7 ± 0.11 ^c^
135	39.2 ± 0.06 ^c^	2.2 ± 0.02 ^b^	34.2 ± 0.13 ^b^	7.6 ± 0.12 ^c^

The values represent mean ± standard deviation (SD), *n* = 5; Means in columns with different letters differ significantly (*p* ≤ 0.05).

**Table 4:** Body weight gain, food intake and food efficiency in rats fed with the control diet and the experimental diets.

Parámetros	Control	N-60	N-200	N-400	N-600
Initial weight (g)	124.8 ± 18.5 ^a^	124 ± 15.8 ^a^	124.7 ± 15.2 ^a^	124.7 ± 15.4 ^a^	124.8 ± 15.7 ^a^
Final weight (g)	334.4 ± 22.5 ^a^	316.7 ± 20.9 ^a^	341.4 ± 21.2 ^a^	322.8 ± 21.8 ^a^	346 ± 34.6 ^a^
Weight gain (g)	209.5 ± 22.6 ^a^	192.7 ± 22.2 ^a^	216.7 ± 16.7 ^a^	198.1 ± 19.3 ^a^	221.2 ± 30 ^a^
Food intake (g)	1075 ± 53.2 ^a^	938 ± 24.2 ^b^	933 ± 23.8 ^b^	975 ± 34.3 ^b^	926 ± 21.1 ^b^
Food efficiency *	0.19 ± 0.01 ^a^	0.2 ± 0.02 ^a,b,c^	0.23 ± 0.01 ^b,c^	0.2 ± 0.01 ^a,b^	0.23 ± 0.03 ^c^

The values represent mean ± SD *n* = 7; Means in rows with different letters differ significantly (*p* ≤ 0.05); * Food efficiency = weight gain (g)/food intake (g).

**Table 5:** Physical and mechanical properties of the femur in rats fed with *O. ficus indica* as calcium source.

Parameters	Control	N-60	N-200	N-400	N-600
Length (cm)	3.65 ± 0.09 ^a^	3.61 ± 0.04 ^a^	3.69 ± 0.07 ^a^	3.65 ± 0.04 ^a^	3.66 ± 0.09 ^a^
Weight (g)	1.03 ± 0.08 ^a,b^	0.95 ± 0.06 ^a^	1.02 ± 0.06 ^a,b^	1.00 ± 0.07 ^a,b^	1.1 ± 0.15 ^b^
Width (mm)	4.31 ± 0.12 ^a^	4.38 ± .23 ^a^	4.22 ± 0.11 ^a^	4.27 ± 0.14 ^a^	4.5 ± 0.29 ^a^
Thickness (mm)	3.18 ± 0.1 ^a,b^	3.05 ± 0.05 ^b^	3.17 ± 0.09 ^a,b^	3.21 ± 0.1 ^a,b^	3.30 ± 0.15 ^a^
Three-point bending test *P*_max_ (N)	98.63 ± 5.2 ^c^	78.58 ± 2.3 ^a^	90.78 ± 1.7 ^b^	98.78 ± 4 ^c^	99.34 ± 2.5 ^c^
Compression test *F*_max_ (N)	610.3 ± 40.6 ^b^	466.4 ± 29.7 ^a^	497.5 ± 46 ^a^	571.2 ± 18.5 ^b^	747.9 ± 97.8 ^c^
*E* (N/mm^2^)	825 ± 78 ^a^	2554 ± 283 ^b^	1304 ± 230 ^a^	2176 ± 367 ^b^	2700 ± 194 ^b^

The values represent mean ± SD *n* = 7; Means in rows with different letters differ significantly (*p* ≤ 0.05). *F*_max_: failure load evaluated by the compression test; *P*_max_: failure load evaluated by three-point bending test.

**Table 6:** Mineral content and Ca/P ratio within the femur of rats fed with control and experimental diets.

Group	Calcium (mg/g)	Phosphorous (mg/g)	Magnesium (mg/g)	Potassium (mg/g)	Ca/P Ratio
Control	385.09 ± 2.2 ^e^	92.81 ± 1.17 ^c^	1.88 ± 0.015 ^b^	0.387 ± 3 × 10^−3 b^	3.2 ^a^
N-60	196.63 ± 3.4 ^a^	90.48 ± 0.37 ^b,c^	1.84 ± 0.021 ^a,b^	0.355 ± 1 × 10^−3 a^	1.7 ^d^
N-200	291.89 ± 8.6 ^b^	87.60 ± 0.40 ^a^	1.81 ± 0.029 ^a^	0.512 ± 7 × 10^−3 b,c^	2.5 ^c^
N-400	333.67 ± 2.3 ^c^	89.85 ± 1.39 ^a,b^	1.87 ± 0.012 ^b^	0.405 ± 1 × 10^−3 b^	2.8 ^b^
N-600	356.18 ± 6.8 ^d^	88.59 ± 0.72 ^a,b^	1.82 ± 0.008 ^a^	0.682 ± 1 × 10^−3 c^	3.1 ^a^

Values represent mean ± SD *n* = 7; Means in columns with different letters differ significantly (*p* ≤ 0.05).

**Table 7:** Correlation coefficients between bone mineral density (BMD) and mechanical, microstructural properties and mineral content within the femur of growing rats.

All Groups (*n* = 35)	*r*	*p*
Mechanical properties		
*F*_max_	0.667	˂0.01
*P*_max_	0.603	˂0.01
Microstructural properties		
Cr.Wi	0.460	˂0.05
Tb.Th	0.466	˂0.01
Tb.Sp	−0.558	˂0.01
Femur mineral content		
Calcium	0.544	˂0.05
Phosphorus	N.S.	N.S.
Potassium	0.654	˂0.05
Magnesium	N.S.	N.S.
Ca/P ratio	0.583	˂0.01

*F*_max_: failure load evaluated by the compression test; *P*_max_: failure load evaluated by three-point bending test; N.S.: Not significant; Tb.Th: trabecular thickness; Tb.Sp: trabecular separation; *r:* Pearson correlation; *p*: significance level.
